# Increased *Plasmodium chabaudi* malaria mortality in mice with nutritional iron deficiency can be reduced by short-term adjunctive iron supplementation

**DOI:** 10.1186/s12936-018-2186-8

**Published:** 2018-01-16

**Authors:** Filip C. Castberg, Lasse Maretty, Trine Staalsoe, Casper Hempel, Erik Clasen-Linde, Lars Hviid, Jørgen A. L. Kurtzhals

**Affiliations:** 1 Centre for Medical Parasitology, Department of Clinical Microbiology, Copenhagen University Hospital (Rigshospitalet), Copenhagen, Denmark; 2 Centre for Medical Parasitology, Department of Infectious Diseases, Copenhagen University Hospital (Rigshospitalet), Copenhagen, Denmark; 3 Centre for Medical Parasitology, Department of Immunology and Microbiology, Faculty of Health and Medical Sciences, University of Copenhagen, Copenhagen, Denmark; 4 Department of Pathology, Copenhagen University Hospital (Rigshospitalet), Copenhagen, Denmark; 5 Present Address: Department of Micro- and Nanotechnology, Technical University of Denmark, Lyngby, Denmark

**Keywords:** *Plasmodium chabaudi* AS, A/J mice, Iron deficiency, Malaria, Hepcidin, FGF23

## Abstract

**Background:**

Iron deficiency is the most widespread nutrient deficiency and an important cause of developmental impairment in children. However, some studies have indicated that iron deficiency can also protect against malaria, which is a leading cause of childhood morbidity and mortality in large parts of the world. This has rendered interventions against iron deficiency in malaria-endemic areas controversial.

**Methods:**

The effect of nutritional iron deficiency on the clinical outcome of *Plasmodium chabaudi* AS infection in A/J mice and the impact of intravenous iron supplementation with ferric carboxymaltose were studied before and after parasite infection. Plasma levels of the iron status markers hepcidin and fibroblast growth factor 23 were measured in animals surviving and succumbing to malaria, and accompanying tissue pathology in the liver and the spleen was assessed.

**Results:**

Nutritional iron deficiency was associated with increased mortality from *P. chabaudi* malaria. This increased mortality could be partially offset by carefully timed, short-duration adjunctive iron supplementation. Moribund animals were characterized by low levels of hepcidin and high levels of fibroblast growth factor 23. All infected mice had extramedullary splenic haematopoiesis, and iron-supplemented mice had visually detectable intracellular iron stores.

**Conclusions:**

Blood transfusions are the only currently available means to correct severe anaemia in children with malaria. The potential of carefully timed, short-duration adjunctive iron supplementation as a safe alternative should be considered.

## Background

Iron deficiency is the most widespread nutrient deficiency, causing considerable developmental impairment in children [1–4], and it is a major cause of anaemia in tropical and low-income countries [5, 6]. However, iron deficiency has also been proposed to protect against malaria [7–10], and there is some evidence that iron supplementation can increase malaria susceptibility [11, 12]. This has made the concept of dietary iron supplementation in areas of stable transmission of malaria parasites somewhat controversial. The issue is complicated by the inherent difficulty in accurately assessing iron status in malaria patients, because all known markers of iron stores are increased by inflammation or are otherwise affected by malaria [13]. This means that severely ill malaria patients might inadvertently be misclassified as iron replete. Concurrent helminth infections, haemoglobinopathies, glucose-6-phosphate-dehydrogenase, and molecular haptoglobin variants can all affect measurements, adding further complexity to epidemiological studies in malaria-endemic areas [14].

To some extent, these difficulties can be overcome in studies of experimental animal models of malaria, as these allow full control over the cause, degree, and alleviation of iron deficiency, as well as control over the timing and clinical consequences of malaria infection in iron-deficient and iron-replete animals. Nevertheless, such studies have also shown conflicting results [15–19].

In the present study, the effect of nutritional iron deficiency and iron supplementation on survival and infection parameters in a murine model of malaria is explored. The focus is on ferric carboxymaltose, which is an effective parenteral iron supplementation that can be administered easily and safely [20–24]. This supplement offers a cost-effective and fast correction of iron deficiency that overcomes the defective absorption of oral iron among parasitaemic recipients, and is without the adverse effects of earlier intravenous preparations.

## Methods

### Experimental animal model

Pathogen-free male A/J mice were purchased from Harland Laboratories (Venray, Netherlands) or Jackson Laboratories (Sacramento, CA, USA). The animals were kept under standard conditions in a closed, ventilated rack system with food/water access ad libitum. Control animals were fed a standard, iron-replete diet (100 mg Fe/kg, Altromin 1319), whereas test animals were fed an iron-deficient diet (5 mg Fe/kg; Altromin C1038, Lage, Germany) to induce iron deficiency anaemia. Haemoglobin levels were monitored weekly until levels were 20% below those in control animals. This was usually achieved in 3–4 weeks.

Some animals were treated daily for 3 consecutive days with i.v. injection (200 μL) of either ferric carboxymaltose (equivalent of 600 μg Fe; Ferinject, Vifor Pharma, Glattbrugg, Switzerland) [25] or ferrous sulfate p.o. (600 μg Fe in 200 μL water). In those experiments, control animals received the same volume of either carboxymaltose i.v. (900 μg) or saline p.o.

For malaria infection, *P. chabaudi* AS-infected erythrocytes (IEs), obtained from frozen stock (originally a gift from David Walliker, University of Edinburgh, UK), were used. After thawing, the parasites were passaged in donor mice before infecting study animals i.p. [26].

A body temperature below 30 °C was used as a humane endpoint for death, and such severely hypothermic animals were killed by cervical dislocation as required by the Inspectorate [27].

### Outcome measurements

Weight, body temperature, haemoglobin level, reticulocytaemia, and asexual parasitaemia were measured daily until malaria symptoms developed. Subsequently, the body temperature and clinical condition of the animals were checked thrice daily.

Body temperatures were measured with an infrared thermometer (845, Testo, Lenzkirch, Germany), with temperatures below 31 °C further assessed by a rectal probe (DM852, Ellab, Hillerød, Denmark), as described [27]. Haemoglobin levels were measured in blood from tail nicks by alkaline haematin D–575 spectrophotometry, as described [28]. Reticulocytaemias and asexual parasitaemias were determined by flow cytometry and assessment of DNA/RNA content of acridine orange-labelled blood as described  [21, 29]. FGF23 was measured using a C-terminal, homologous two-site enzyme-linked immunosorbent assay (ELISA), catalogue number #60-6300 (Immutopics Inc, CA, USA). Hepcidin (hepc-1) was detected with a solid-phase ELISA, based on the principles of competitive binding, catalogue number SKU# HMC-001, Intrinsic Lifesciences, CA, USA.

### Histochemistry

In some experiments, mice were censored to allow harvesting of organs at different time points during infection. These animals were anesthetized using fentanyl/fluanisone (Hypnorm) and Midazolam i.p., immediately perfused with saline and subsequently perfusion fixated using formaldehyde 4%, pH 7.4, and killed in the process. Livers and spleens were initially incubated in formaldehyde (24 h) and then stored in 70% ethanol. All organs were embedded in paraffin, sectioned (2 µm), and stained using Haematoxylin–Eosin and Perls’ Prussian Blue iron staining. Sections were evaluated by light microscopy. The examiner was blinded to treatment group affiliations.

### Statistics

Statistical analyses were done in SAS v. 9.4 (SAS Institute, NC, USA). The log-rank test was used to assess the statistical significance of survival effects, and continuous outcome measures were analysed using a mixed-effects model (Autoregressive, order 1) with individual mice and treatment groups included as repeated and fixed effects, respectively. Hepcidin and FGF23 levels were analysed using log-transformed values in Satterthwaite’s approximate t test and ANOVA. P values < 0.05 were considered statistically significant.

## Results

### Determination of appropriate inoculum size

**Fig. 1:**
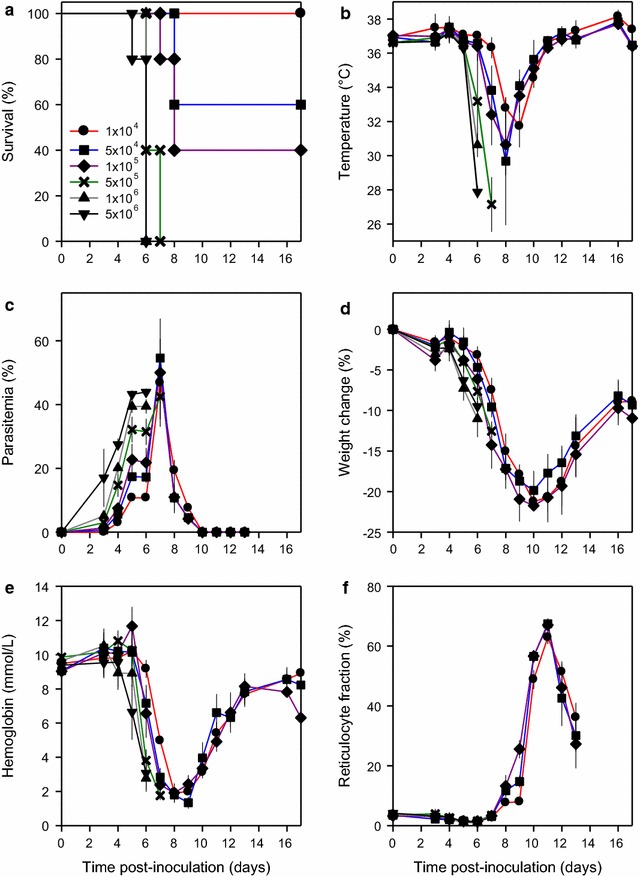
Establishment of inoculum size. Clinical course in mice (5 per group) infected intraperitoneally with *P. chabaudi* AS-IEs on Day 0 [1 × 10^4^ (black circle red line), 5 × 10^4^ (black square blue line), 1 × 10^5^ (black diamond purple line), 5 × 10^5^ (black times green line), 1 × 10^6^ (black triangle grey line), 5 × 10^6^ (black inverse triangle black line)]. Panels depict **a** survival, **b** temperature, **c** parasitaemia, **d** weight change, **e** haemoglobin, and **f** reticulocyte fraction. Group means (symbols) and SD (error bars; **b**–**f**) are indicated

To establish an appropriate inoculum size that would result in mortality in some but not all infected control animals, groups of mice i.p. were inoculated with increasing numbers of IEs (1 × 10^4^ − 5 × 10^6^) (Fig. 1). In all groups receiving > 1 × 10^4^ IEs, some or all the mice died or had to be killed (due to legal requirements, see Methods) before the end of the experiment (Fig. 1a). The body temperature of surviving animals decreased from Day 5 to Day 8–9 post-inoculation, followed by gradual recovery, until temperatures had returned to normal about Day 12 (Fig. 1b). Parasitaemias increased until Day 7 post-infection, followed by a decline to very low levels by Day 10 (Fig. 1c). Changes in weight (Fig. 1d), haemoglobin concentration (Fig. 1e), and reticulocytaemia (Fig. 1f) were essentially independent of inoculum size (Fig. 1d). Based on these data, inocula of 1 × 10^4^ or 1 × 10^5^ IEs were used in all subsequent experiments.

### Carboxymaltose treatment reduces malaria mortality in iron-replete animals

**Fig. 2:**
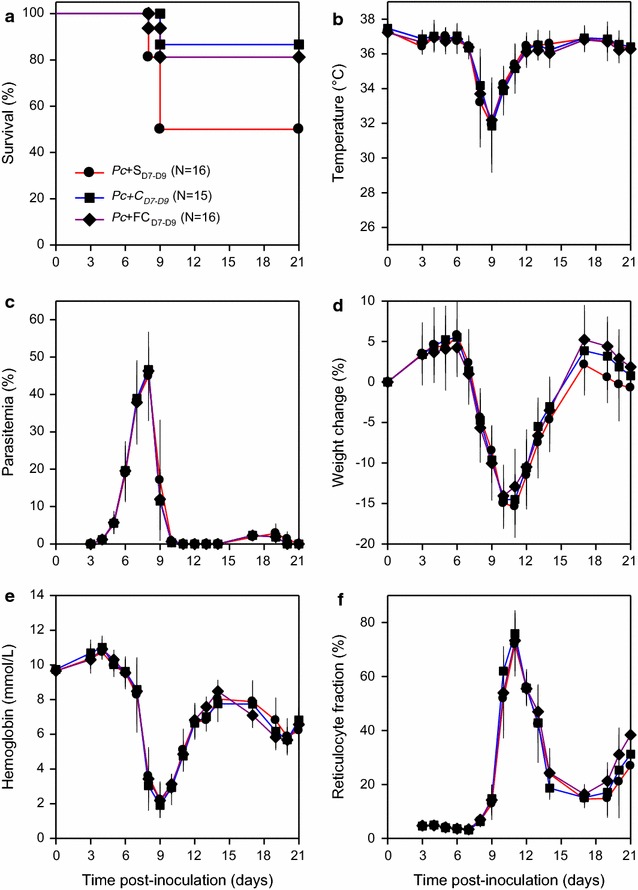
Effect of ferric carboxymaltose on *Plasmodium chabaudi* infection. Clinical course in mice infected intraperitoneally with 1 × 10^5^ *P. chabaudi* AS-IEs on Day 0, and treated with daily (Days 7–9 post-inoculation only) i.p. injection of saline (black circle red line), carboxymaltose (black square blue line), or ferric carboxymaltose (black diamond purple line). Panels depict **a** survival, **b** temperature, **c** parasitaemia, **d** weight change, **e** haemoglobin, and **f** reticulocyte fraction. Initial group sizes, group means (symbols) and SD (error bars; **b**–**f**) are indicated

To assess if the planned iron supplementation regimen would by itself affect the course of malaria even in iron-replete animals, *P. chabaudi* AS-infected (1 × 10^5^ IEs i.p.) mice were treated once daily from Day 7 to Day 9 post-infection with saline, carboxymaltose, or ferric carboxymaltose (Fig. 2). Mortality was lower in the groups treated with ferric carboxymaltose (mortality 19%; P = 0.07) or carboxymaltose (13%; P = 0.03), compared to the saline-treated group (mortality 50%) (Fig. 2a). In contrast, body temperatures (Fig. 2b), parasitaemias (Fig. 2c), weights (Fig. 2d), haemoglobin levels (Fig. 2e), and reticulocytaemias (Fig. 2f) were essentially identical among all three groups. As mortality was similar in the ferric carboxymaltose- and carboxymaltose-treated groups (P = 0.7), these results indicate that the effect on mortality mediated by the carbohydrate and/or its degradation products was not affected by its Fe-complex. The reduced mortality could reflect reduced hypoglycaemia in both the ferric carboxymaltose- and carboxymaltose-treated groups [30, 31]. Therefore, carboxymaltose-treated animals were used as controls in the experiments where the effect of ferric carboxymaltose was tested in iron-deficient animals.

### Nutritional iron deficiency aggravates the course of malaria

**Fig. 3:**
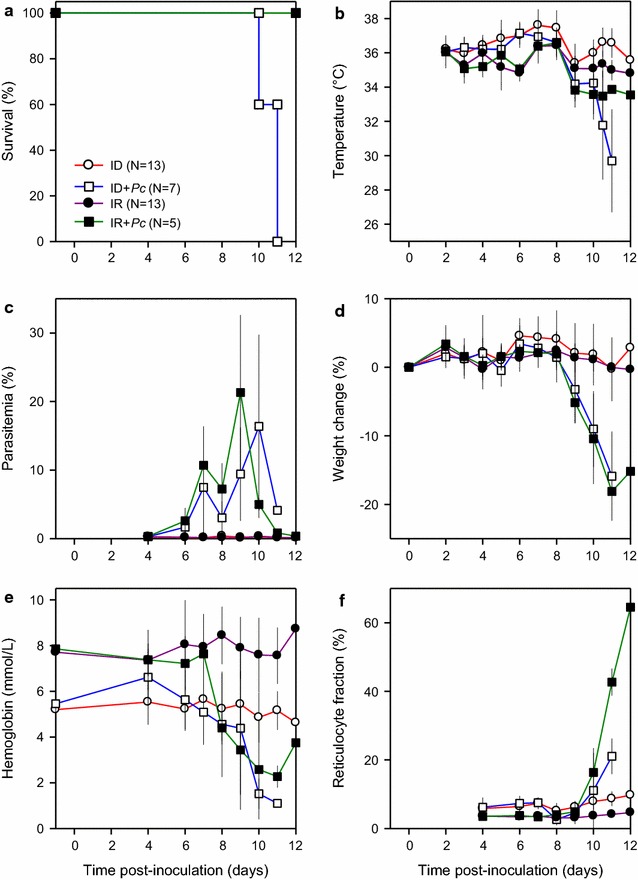
Effect of nutritional anaemia on *Plasmodium chabaudi* infection. Clinical course in uninfected (white circle red line, black circle purple line) and *P. chabaudi* AS-infected (1 × 10^4^ IEs i.p., white square blue line, black square green line), iron-deficient (white circle red line, white square blue line) or iron-replete mice (black circle purple line, black square green line). Panels depict **a** survival, **b** temperature, **c** parasitaemia, **d** weight change, **e** haemoglobin, and **f** reticulocyte fraction. Initial group sizes, group means (symbols) and SD (error bars; **b**–**f**) are indicated. For clarity, data regarding non-infected control animals (100% survival) are not shown in **a**

To study the effect of nutritional anaemia on the clinical course of *P. chabaudi* AS infection, iron-replete and iron-deficient animals were infected (1 × 10^4^ IEs i.p.), and the course of infection was observed (Fig. 3). Although this inoculum was established as non-lethal in iron-replete animals (Fig. 1a), all the infected iron-deficient animals in this experiment died or had to be killed (Fig. 3a). All uninfected animals survived, whether iron-deficient or iron-replete. In the infected animals, temperatures dropped slightly (iron-replete animals) or precipitously (iron-deficient animals) from around Day 6 (Fig. 3b), although parasitaemias (Fig. 3c) and weights (Fig. 3d) developed similarly in iron-deficient and -replete infected mice (Fig. 3c). Interestingly, infected iron-replete and iron-deficient animals became similarly and severely anaemic (Fig. 3e), despite their very different haemoglobin levels at the outset of the experiment (Fig. 3e). Substantial reticulocytaemia was observed in infected animals (Fig. 3f). In conclusion, nutritional iron deficiency made the mice more susceptible to *P. chabaudi* AS infection and led to a fatal outcome of a normally non-lethal infection. Iron supplementation had no effect on parasitaemia, which was similar in all groups (Fig. 3c). This study is a refinement from two pilot experiments with similar results using iron-deficient infected saline controls.

### The protective effect of iron supplementation critically depends on when it is given

**Fig. 4:**
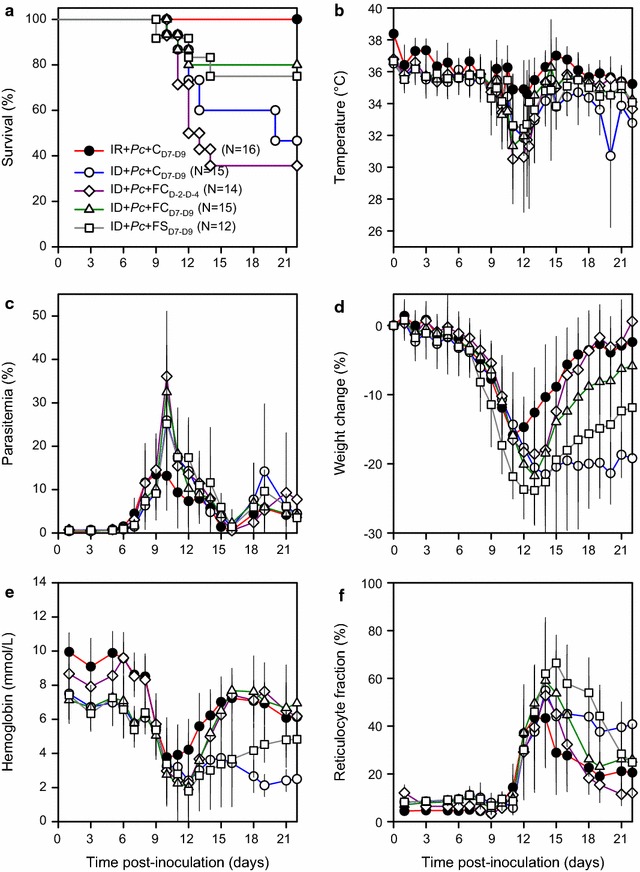
Effect of iron supplementation on *Plasmodium chabaudi* infection. Clinical course in *P. chabaudi* AS-infected (1 × 10^4^ IEs i.p.), iron-replete (black circle red line) or iron-deficient mice (white circle blue line, white diamond purple line, white triangle green line, white square grey line), treated on Day −4 to Day −2 before infection only (white diamond purple line) or on Day 7 to Day 9 post-infection only (black circle red line, white circle blue line, white diamond purple line, white triangle green line) with carboxymaltose i.p. (black circle red line, white circle blue line), ferric carboxymaltose i.p. (white diamond purple line, white triangle green line), or ferrous sulfate p.o. (white square grey line). Panels depict **a** survival, **b** temperature, **c** parasitaemia, **d** weight change, **e** haemoglobin, and **f** reticulocyte fraction. Initial group sizes, group means (symbols) and SD (error bars; **b**–**f**) are indicated

Next, it was tested whether iron supplementation could ameliorate the observed aggravated course of *P. chabaudi* AS infection in iron-deficient mice (Fig. 4). To that end, the course of infection in groups of iron-deficient animals was compared following inoculation as above (1 × 10^4^ IEs i.p.). The following regimens of iron supplementation were tested: (i) ferric carboxymaltose i.v. from 4 to 2 days before parasite inoculation, (ii) ferric carboxymaltose i.v. from 7 to 9 days after inoculation, and (iii) ferrous sulfate p.o. from 7 to 9 days after inoculation. Iron-replete and iron-deficient animals receiving carboxymaltose i.v. from 7 to 9 days after inoculation were included as controls.

Mortality was observed in all groups except the iron-replete control group (Fig. 4a). Mortality among the iron-deficient control animals was substantial (62%) but lower than the uniform mortality seen in the previous experiment, probably due to the protective effect of carboxymaltose used here. Iron supplementation on Day 7 to Day 9 post-inoculation reduced mortality, whether supplementation was given as ferric carboxymaltose (20% mortality; P = 0.05) or as ferrous sulfate (31% mortality; P = 0.26). However, in striking contrast, ferric carboxymaltose given to iron-deficient animals a few days prior to parasite inoculation had no protective effect (mortality 62%, P = 0.64), and in fact seemed to cause a more rapid onset of fatal pathology compared to the control mice.

Body temperatures (Fig. 4b), parasitaemias (Fig. 4c), and reticulocytaemias (Fig. 4f) developed similarly in all groups of iron-deficient mice regardless of the supplementation regimens. With respect to weights, mice in all groups lost weight in a similar way until about Day 11 (Fig. 4d). However, the normal recovery of body weight following the parasitaemic crisis seen in the iron-replete, carboxymaltose-treated control mice was completely absent from the iron-deficient control mice treated the same way. Iron-supplementation of iron-deficient mice allowed the mice to regain some of the malaria-induced weight loss, in particular when treatment was given before the parasite infection (Fig. 4d). Ferric carboxymaltose appeared more efficient than ferrous sulfate in that respect. Haemoglobin levels (Fig. 4e) were initially higher among the iron-deficient mice that received ferric carboxymaltose prior to inoculation compared to the other iron-deficient mice, and were similar to those of iron-replete animals from Day 6 onwards. In all study groups, haemoglobin levels fell from about Day 5 to about Day 11, followed by recovery in the iron-replete controls and the iron-supplemented, iron-deficient mice. This was most pronounced among mice receiving ferric carboxymaltose, in which recovery was similarly to that in the replete animals. No recovery in haemoglobin levels was seen among the non-supplemented iron-deficient animals.

In conclusion, iron supplementation can alleviate malaria-related mortality in iron-deficient mice, but the protective effect depends critically on the timing of the supplementation. This study is a refinement from a pilot experiment with similar results using iron-deficient infected saline controls.

### Serological iron status markers and haematopoietic histopathology in malaria-infected mice

**Fig. 5:**
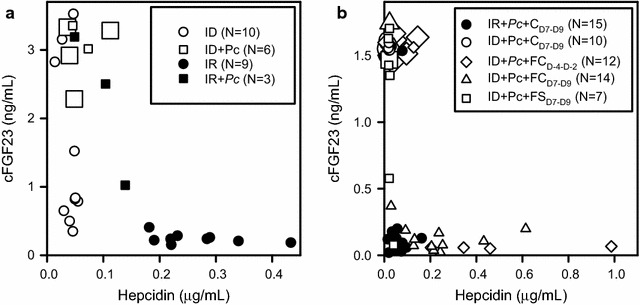
Relation between outcome and plasma levels of hepcidin and FGF23. **a** Plasma levels in individual iron-replete (black circle, black square) and iron-deficient (white circle, white square), uninfected (black circle, white circle) or *P. chabaudi* AS-infected (black square, white square) mice. **b** Plasma levels in individual *P. chabaudi* AS-infected iron-replete (black circle) and iron-deficient (white circle, white diamond, white triangle, white square) mice. The mice were treated with carboxymaltose Day 7 to Day 9 post-infection (black circle, white circle), with ferric carboxymaltose Day 4 to Day 2 before infection (white diamond) or Day 7 to Day 9 post-infection (white triangle), or with ferrous sulfate Day 7 to Day 9 post-infection (white square). Animals that died or had to be killed are shown with large symbols, whereas animals that survived are shown with small symbols

Hepcidin is a peptide hormone that negatively regulates dietary iron absorption. Hepcidin levels increase in response to inflammation, but are decreased in anaemia [32, 33]. The hormone has anti-inflammatory properties in mice [34]. Fibroblast growth factor 23 (FGF23) is a bone-regulating hormone that has been reported to correlate with iron status irrespective of inflammatory status, although the evidence is equivocal [35, 36]. To assess the utility of these markers in the model, their levels were measured in plasma samples obtained at different time points from the animals used in the experiments reported above (Fig. 5). Hepcidin levels in uninfected mice fell in two clear groups depending on iron status (Fig. 5a, P = 0.009). Contrary to expectations, only four of ten uninfected, iron-deficient mice had elevated FGF23, however, a significant difference was still detected between iron-deficient and iron-replete controls (Fig. 5a, P = 0.009). Hepcidin levels were clearly driven by anaemia and tended to be lower in infected than uninfected iron-replete mice (Fig. 5a, P = 0.07). A trend towards high FGF23 and low hepcidin in all fatal cases was noticed (Fig. 5, large symbols). In order to substantiate this and to study the effect of treatment regimens on hepcidin and FGF23, an experiment with infected mice only was conducted. Ferric carboxymaltose-treated mice had significantly higher hepcidin levels than the other groups (Fig. 5b, P = 0.0001). As in the former experiment (Fig. 5a), iron-depleted mice showed a dichotomy in FGF23 levels, and all fatal cases had high FGF23 (Fig. 5b). Across the iron deficient mice, fatal cases had significantly higher FGF23 than non-fatal cases (P = 0.0001).

**Fig. 6:**
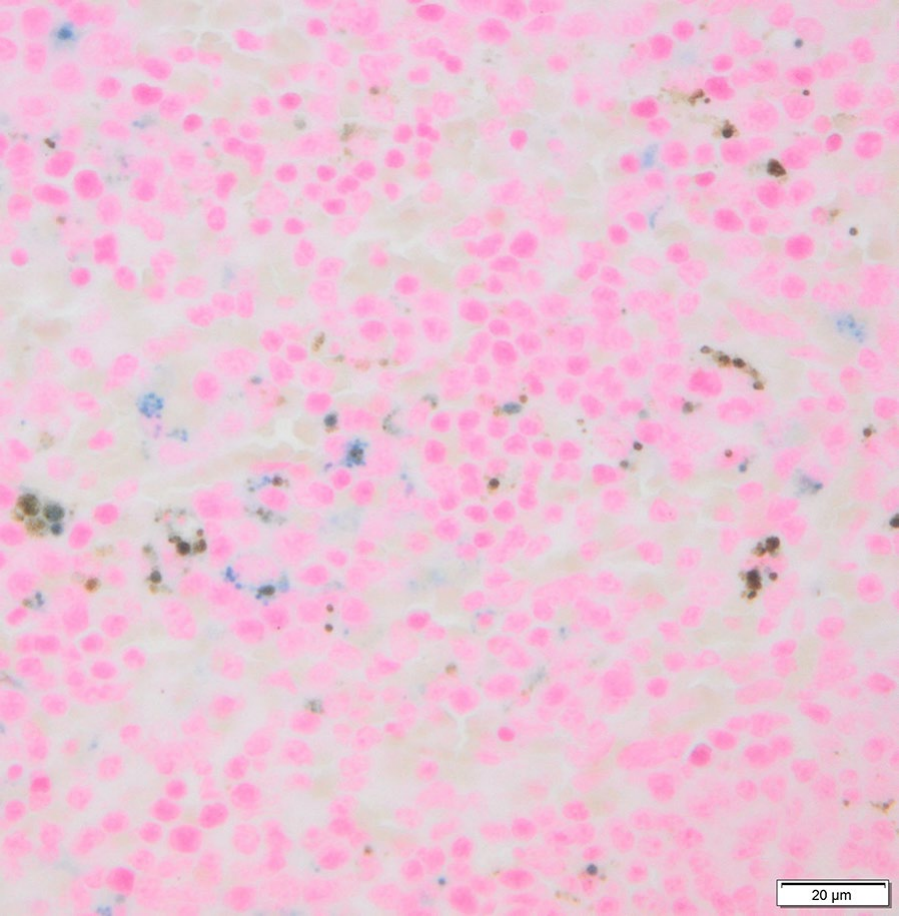
Intracellular iron stores in the spleen. Perl’s Prussian Blue iron stain of the spleen from an iron deficient mouse that received intravenous ferric carboxymaltose. Iron deposits within macrophages appear bright blue. Five spleens of surviving animals randomly selected from every group (n = 25) were examined at the end of the experiment (Day 22), when the mice were killed. All iron-replete mice and ferric carboxymaltose-supplemented iron-deficient mice had intracellular iron stores in the spleen, whereas this was not seen in any of the carboxymaltose- or ferrous sulfate-treated mice (not shown) except scarce amounts of iron in 1 of 5 iron-deficient mice that received carboxymaltose Day 7 to Day 9 post-infection

Also, the livers and spleens of surviving animals at the end of the experiments (Day 22) were examined, when remaining mice were killed. All mice showed evidence of extramedullary haematopoiesis in the spleen. Iron-replete mice and ferric carboxymaltose-supplemented iron-deficient mice had intracellular iron stores in the spleen (Fig. 6), whereas this was not seen in carboxymaltose- or ferrous sulfate-treated mice except in one carboxymaltose-treated mouse with scarce iron stores. Minor areas of microsteatosis and necrosis were observed in livers, most prominently in animals that died of malaria, but the severity of these changes was not sufficient to be considered the cause of death, and no attempts were made to quantify the lesions.

## Discussion

Iron is an essential nutrient, but its redox properties makes it potentially cytotoxic. Levels of non-transferrin-bound iron ions are therefore tightly regulated by hormones such as hepcidin. In the face of infection, a balance must furthermore be struck between the host need for iron and the potential benefits of minimizing the availability of this nutrient to invading microorganisms [37]. This has created substantial controversy regarding the role of iron status in host health versus susceptibility to important infectious diseases such as malaria, and the relative benefits and detriments of correcting iron deficiency by dietary supplementation in malaria-endemic areas [38].

In the present study, nutritional iron deficiency was found to seriously aggravate *P. chabaudi* AS infection in A/J mice. Thus, an inoculum size that normally produced a self-limiting infection in iron-replete animals caused substantial mortality among iron-deficient mice. The obligation to kill animals with a body temperature below 30 °C (to minimize suffering) may have led to some overestimation of mortality, but these animals were all clinically assessed moribund, why the implementation of this humane endpoint did not markedly affect the outcome of the experiments or the conclusions that can be drawn. As such, the presented data are at variance with experimental animal studies [15, 18] and human field studies that have indicated that iron deficiency can protect against malaria [7, 9, 10, 39].

Both altered iron bioavailability and erythrocyte physiology have been implicated as mechanisms underlying the putative protective effect of iron deficiency [19, 40]. Clark et al. speculated that iron deficiency might induce a reduction in the erythropoietic rate and the synthesis of microcytic, iron-deficient erythrocytes unsuitable as host cells for the parasites [41]. In contrast, Matsuzaki-Moriya et al. found that iron deficiency anaemia did not in itself have an effect on parasite growth, and instead suggested that parasitized erythrocytes from malaria patients with iron deficiency anaemia are more susceptible to phagocytosis than control cells  [18]. However, there have also been studies challenging the protective effect of iron deficiency. Thus, Lelliott et al. found that mice carrying a missense mutation in the transferrin receptor 1 gene, which renders them anemic, showed higher *P. chabaudi* parasitaemias and mortality than wildtype mice [19], similar to the findings in the present study. Furthermore, iron is required for normal immune function, and iron deficiency anaemia is a risk factor in acute lower respiratory tract infection and in acute otitis media [42, 43].

With respect to the malaria consequences of correcting iron deficiency, this study documents a markedly beneficial effect of short-term iron supplementation on the survival of iron-deficient, *P. chabaudi* AS-infected A/J mice. The iron supplementation did not lead to increased parasitaemia, in agreement with our previous safety study [21]. On the other hand, it also did not reduce parasitaemia, thus suggesting that the improved survival rates were not due to a direct effect on parasites but rather due to an improved host response. In the initial survival experiment, using a lethal inoculum (Fig. 2), the effect on survival might be ascribed to reversal of hypoglycaemia [30, 31], since additional experiments showed a similar survival effect after administration of dextrose (unpublished data). However, in iron-deficient mice infected with low inoculum, the survival mediated by ferric carboxymaltose was not due to a glycemic effect, since carboxymaltose did not rescue the mice. Further studies are needed to dissect the host effects causing vulnerability to *P. chabaudi* in iron deficient mice, and how these are affected by ferric carboxymaltose.

This study’s results also indicate that the temporal relationship between iron deficiency and parasitaemia is important. Thus, restoration of iron stores immediately prior to infection did not improve survival in our hands, but rather tended to hasten fatal outcomes. These findings are in line with those of Clark et al., which led them to suggest a period of vulnerability during the transition from iron deficiency to iron replete status [41]. Thus, a ‘window of opportunity’ may exist, where adjunct intravenous iron supplementation would be beneficial. In that context, it may be worth noting that Chang et al. found that early reticulocytosis increased morbidity in a murine malaria model, whereas late reticulocytosis had the opposite effect [44]. Furthermore, the study’s findings suggest that iron supplementation should be administered over as short a period as possible in order to reduce the period of vulnerability, and preferably while taking precautions against malaria and other infections. In this connection, it should be noticed that the protective effect of the long-acting partner drug (e.g. lumefantrine or piperaquine) may shield against a temporary risk of malaria if i.v. iron is administered concomitantly with artemisinin-based combination therapy.

The fact that this study was based on pilot experiments, and thus had clearly predefined hypotheses, supports the conclusions regarding an effect of iron deficiency as well as of ferric carboxymaltose therapy on severity of *P. chabaudi* infections. However, the data should be interpreted with caution.

Previous clinical studies of iron supplementation have shown conflicting results. While some findings suggest that supplementation may be unwise [12, 45], others reported no negative effect on malaria susceptibility of iron supplementation [46–48]. Recent meta-analyses indicate that iron supplementation does not increase the risk of malaria or malaria-related death, when regular surveillance and treatment services are provided [49, 50].

This study’s results do not support the proposition by Braithwaite et al. that FGF23 can be used as a marker of iron status in patients exposed to malaria [35], which would be an improvement over hepcidin, which is both affected by and itself affecting inflammation [32, 34]. In particular, mice with nutritional iron deficiency could have either high or low FGF-23, indicating that this is not a useful marker of iron deficiency during malaria. Furthermore, all dying mice had severe anaemia, low hepcidin levels and high FGF23 levels. The data do not allow a conclusion on whether the high FGF23 levels in these mice were primarily caused by anaemia or inflammation [36]. The combination of high FGF23 and low hepcidin levels could possibly serve as a marker of severity of malaria and a predictor for outcome. Further research on how iron-regulating hormones affect survival in complicated malaria is warranted.

## Conclusion

This study provides evidence that nutritional iron deficiency is a risk factor in malaria and suggests that the risk can be alleviated by carefully timed, short-duration adjunct iron supplementation. Intravenous iron treatment in low-resource settings may not be easily implemented, but considering that the only currently available adjunctive treatment for severe malarial anaemia is blood transfusion, with its accompanying risks of infection and anaphylactic reactions, it may nevertheless be the better and safer alternative.
